# Elevation of NT-proBNP Levels in Pediatric and Young Adult Hematopoietic Stem Cell Transplant Patients with Endotheliopathy

**DOI:** 10.3390/pediatric16040080

**Published:** 2024-10-30

**Authors:** Kimberly Uchida, Xiaomeng Yuan, Jennifer McArthur, Rebekah Lassiter, Haitao Pan, Dinesh Keerthi, Katherine Tsai, Yvonne Avent, Melissa Hines, Hugo R. Martinez, Amr Qudeimat, Saad Ghafoor

**Affiliations:** 1 Division of Pediatric Critical Care Medicine, St. Jude Children’s Research Hospital, 262 Danny Thomas Place, MS 734 Room IA3309, Memphis, TN 38105, USA; jennifer.mcarthur@stjude.org (J.M.); yvonne.avent@stjude.org (Y.A.); melissa.hines@stjude.org (M.H.); 2 Division of Pediatric Critical Care Medicine, University of Tennessee Health Science Center, 50 N. Dunlap, 370R, Memphis, TN 38103, USA; 3 Department of Biostatistics, St. Jude Children’s Research Hospital, 262 Danny Thomas Place, MS 768 Room S2001, Memphis, TN 38105, USA; xiaomeng.yuan@stjude.org (X.Y.); haitao.pan@stjude.org (H.P.); 4 Department of Bone Marrow Transplantation & Cellular Therapy, St. Jude Children’s Research Hospital, 262 Danny Thomas Place, MS 321 Room I4112, Memphis, TN 38105, USA; dinesh.keerthi@stjude.org (D.K.); amr.qudeimat@stjude.org (A.Q.); 5 Division of Pediatric Cardiology, University of Tennessee Health Science Center, 49 N. Dunlap St. 3rd Floor, Memphis, TN 38103, USAhugo.martinez@austin.utexas.edu (H.R.M.); 6 Kaiser Permanente, General Pediatrics, 12815 Heacock Street, Moreno Valley, CA 92553, USA; 7 Division of Internal Medicine and Pediatric Cardiology, Dell Medical School, The University of Texas at Austin, 4910 Mueller Blvd., Suite 102, Austin, TX 78723, USA

**Keywords:** hematopoietic stem cell transplantation (HSCT), NT-proBNP, endotheliopathy, diffuse alveolar hemorrhage (DAH), sinusoidal obstruction syndrome (SOS), thrombotic microangiopathy (TMA), pediatric intensive care unit (PICU)

## Abstract

Background/Objectives: Hematopoietic stem cell transplantation (HSCT) in pediatric and young adult (YA) patients can lead to endotheliopathy, such as thrombotic microangiopathy (TMA), sinusoidal obstruction syndrome (SOS), and diffuse alveolar hemorrhage (DAH). Natriuretic peptides have been studied as markers of endotheliopathy and critical illness. We hypothesized that an elevation in NT-proBNP was associated with the development of endotheliopathy (DAH, SOS, or TMA) in the first 100 days following HSCT in pediatric and YA patients. Methods: IRB-exempt status was obtained. This retrospective case–control study reviewed HSCT at our institution from 2016 to 2020. Cases were selected based on an endotheliopathy diagnosis in the first 100 days after HSCT. Cases were matched with controls. Baseline and near-event NT-proBNP levels were compared between cases and matched controls. The effect of NT-proBNP levels on developing endotheliopathy was estimated using conditional logistic regression. Results: Sixty-two patients were included (31 cases, 31 controls). Near-event NT-proBNP was significantly higher in cases compared to controls (median: 473 vs. 187 pg/mL, *p* = 0.03, Wilcoxon rank–sum test), in contrast to comparison in baseline NT-proBNP (median: 86 vs. 86 pg/mL, *p* = 0.51). After adjusting for covariates, an association between near-event NT-proBNP and odds of developing endotheliopathy did not achieve statistical significance. However, trends from most common transplant indications suggested an association between an elevated near-event NT-proBNP level and endotheliopathy, particularly in acute lymphoblastic leukemia (ALL) patients. Conclusions: NT-proBNP should be studied further as a biomarker for endotheliopathy in pediatric and YA patients undergoing HSCT. This may be particularly relevant for patients undergoing HSCT for ALL.

## 1. Introduction

Endotheliopathy is dysfunction of the endothelium and plays a crucial role in various complications associated with hematopoietic stem cell transplantation (HSCT) in pediatric and young adult patients. These complications can include diffuse alveolar hemorrhage (DAH), sinusoidal obstruction syndrome (SOS), and thrombotic microangiopathy (TMA), among others [1,2,3,4,5,6]. The presence of endotheliopathy can lead to organ dysfunction, the requirement for critical care, and even mortality in these patients [7,8]. It is important for healthcare providers to remain vigilant for signs and symptoms of endotheliopathy and its associated complications in pediatric and young adult patients undergoing HSCT. Early recognition and intervention may help in managing these serious conditions, and hence have the potential to improve patient outcomes. While research is ongoing in the discovery of biomarkers that could help predict endothelial dysfunction in patients receiving HSCT, a specific marker or set of markers has not yet been shown to be fully predictive of such injury [3,5,6].

It is interesting to note the role of B-type natriuretic peptides such as NT-proBNP and BNP in the context of HSCT. These markers are easily obtained in most clinical laboratories and are typically thought of as markers associated with left ventricular dysfunction and cardiac failure [9]. The elevation of these markers following HSCT in adult patients is often attributed to subclinical myocardial toxicity from preparative regimens or prior chemotherapy, even in the absence of clinical or echocardiographic changes [10,11,12,13,14,15,16,17,18]. In pediatric and young adult patients, it is also common practice to monitor NT-proBNP or BNP following HSCT to potentially detect cardiac toxicity. Interestingly, while changes in NT-proBNP levels occur during the peri-transplant and post-transplant time period, studies have not definitively linked NT-proBNP elevation to cardiac outcomes in this specific population [19,20,21,22]. These findings suggest that while B-type natriuretic peptides can serve as valuable markers for cardiac function assessment in HSCT patients, their relationship to clinical outcomes, especially in the pediatric and young adult population, may be more complex than simply a marker of cardiac dysfunction.

BNP has been shown to be an independent risk factor for ICU mortality in adult patients receiving HSCT [23]. Elevated levels of BNP have been associated with increased critical care utilization in pediatric HSCT patients and have also been linked to higher mortality rates in pediatric HSCT patients requiring care in the pediatric intensive care unit (PICU) [24,25]. These studies highlight the potential utility of B-type natriuretic peptides as prognostic markers in HSCT settings, both in pediatric and adult populations. The associations between BNP elevation and adverse outcomes underscore the importance of monitoring B-type natriuretic peptides in HSCT patients to identify those at a higher risk for complications and potentially intervene earlier to improve outcomes.

The relationship between B-type natriuretic peptides (BNP and NT-proBNP) and inflammation is multifaceted. Although ventricular stress is thought to be the most important factor for BNP or NT-pro-BNP secretion, there is evidence that B-type natriuretic peptides are also upregulated with inflammation in cardiac myocytes and other cell types [26,27,28]. Additionally, these peptides have been studied as markers of endotheliopathy in peripheral artery disease and coronary artery disease [28,29]. Given that clinical cardiac toxicity is rare in children undergoing HSCT despite the observed correlation between BNP elevation and critical care utilization and mortality in pediatric patients, we sought to evaluate NT-proBNP as a potential biomarker of endotheliopathy which is more commonly seen than traditional cardiac toxicity [22].

The assessment of endotheliopathy through biomarkers such as B-type natriuretic peptides could provide insight into vascular health and potential cardiovascular risk in HSCT patients, beyond traditional cardiac function assessment. The association between BNP elevation and sinusoidal obstruction syndrome (SOS) in adults has been documented in the literature [30]. However, there is limited research on this relationship in the pediatric population. To address this gap, we hypothesized that an elevation in NT-proBNP is associated with the development of endotheliopathy syndromes such as DAH, SOS, and TMA in the first 100 days following HSCT in children and young adults. We submit our results from a retrospective case–control study at a quaternary pediatric cancer hospital. Our study aimed to provide insight into the utility of NT-proBNP as a biomarker of endothelial dysfunction and development of specific post-transplant complications in children and young adults.

## 2. Materials and Methods

**Figure 1:**
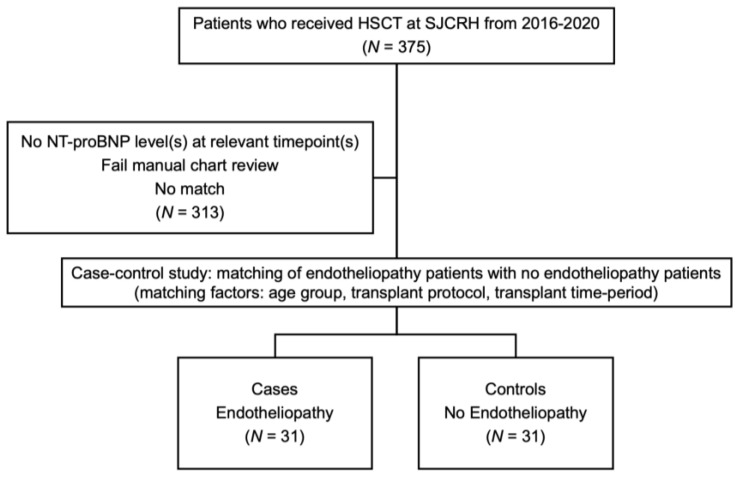
Study methodology. Abbreviations: HSCT, hematopoietic stem cell transplantation; SJCRH, St. Jude Children’s Research Hospital.

This was a retrospective single-center case–control study conducted at St. Jude Children’s Research Hospital. IRB-exempt status was obtained prior to the initiation of the study (IRB number 22-1131). All allogenic or autologous hematopoietic cell transplants at St. Jude Children’s Research Hospital between 2016 and 2020 were initially reviewed (Figure 1).

Cases were then selected based on the diagnosis of DAH, SOS, or TMA in the first 100 days following HSCT. This was performed using a local database. The database was created for clinical and quality improvement purposes to track transplant complications, including DAH, SOS, or TMA. DAH was diagnosed based on bronchioalveolar lavage findings. SOS and TMA were diagnosed using prior published criteria [31,32]. Complications were recorded as per the National Cancer Institute (NCI) Common Terminology Criteria for Adverse Events (CTCAE). All adverse events were reviewed by the attending physicians prior to inclusion in the database.

Potential cases were verified by our study team via a chart review. Patients that did not receive a diagnosis of TMA, SOS, or DAH during the first 100 days following HSCT were excluded as cases but were available for potential use as the controls. Patients that were labeled as only possible, probable, potential, or evolving cases of DAH, SOS, or TMA were excluded for use as cases or controls. Additionally, patients were excluded from selection as cases or controls if the baseline and/or near-event NT-proBNP levels were not available.

Cases were matched with the controls. The matching factors included transplant protocol, age group (<2 years of age, 2–10 years of age, and >10 years of age), and time-period of the transplant (day of transplant between 1 January 2016, and 31 December 2018, or day of the transplant between 1 January 2019, and 31 December 2020). If more than one potential control met matching criteria for any given case, computer randomization was utilized to select the control. If cases did not have a control with adequate matching factors, they were removed from the pool of cases. There were two patients that were noted to have received a diagnosis of SOS that both received tandem transplants. Hence, each of these patients were counted as one case, and the first 100 days following the first HSCT were considered for the purpose of this study. The controls were verified to be true controls by a chart review. This resulted in one additional control being excluded for diagnosis of idiopathic pneumonia syndrome discovered during the chart review.

Baseline NT-proBNP level, echocardiogram data, and renal function by estimated glomerular filtration rate (eGFR) on the closest day prior to transplant were obtained for the cases and controls. Near-event NT-proBNP and echocardiogram data were obtained for the cases. Near-event was defined as the NT-proBNP level and echocardiogram on the day closest to but preceding the day of diagnosis of the event (diagnosis of DAH, SOS or TMA). If data points were not available between the time of transplant and the time of the event, then the time point closest to but after the event was used. These data points were obtained by a manual chart review. For the controls, data extracts from the day after transplant that correlated to the same day after transplant as the matched case’s day of event (diagnosis of endotheliopathy) was used as the near-event timepoint. In the rare case that there was not an echocardiogram available after the baseline echocardiogram, we searched for the first available echocardiogram after the transplant, even if it was after 100 days following HSCT. The ejection fraction was measured via the M-mode. The status of pulmonary hypertension was assessed by indirect measures of tricuspid regurgitation gradient and qualitative assessment.

Demographic and clinical characteristics were compared between the case (N = 31) and control (N = 31) groups using the exact Pearson Chi-square test for the categorical variables and the Wilcoxon rank–sum test for the numeric variables. Based on the comparison results and clinical relevance, a series of conditional logistic regression models were constructed to estimate the effect of the near-event NT-proBNP level on the odds of developing endotheliopathy within the first 100 days following HSCT, controlling for different subsets of covariates. The primary model used the baseline and near-event NT-proBNP level, baseline and near-event ejection fraction, and transplant number as covariates. The secondary model further accounted for potential confounding factors including transplant indication, and age difference (difference between age at transplant and the mean age of the corresponding age grouping) in addition to covariates utilized in the primary model. Akaike information criterion (AIC) scores were calculated for the models. The estimated odds ratio (OR) and 95% Wald confidence interval (CI) were reported for the covariates from the models. The statistical significance cut-off was a *p*-value of 0.05 for all analyses in the study. We used SAS 9.4 to conduct statistical analyses.

## 3. Results

### 3.1. Demographic and Baseline Clinical Characteristics

There was a total of 375 hematopoietic stem cell transplants performed between 2016 and 2020 available for review in the database. The study included 31 cases and 31 matched controls after the application of inclusion and exclusion criteria. Demographic and baseline clinical characteristics are demonstrated in Table 1. Most of the included patients (44, 71%) were between 2 and 10 years of age. Forty-two percent of the patients were female. Fifty-two percent of the patients received HSCT between 1 January 2019 and 31 December 2020. The included patients underwent haploidentical (24, 39%), autologous (20, 32%), matched unrelated donor (12, 19%), and matched sibling donor (6, 10%) HSCT. For most of the patients included, this was the first HSCT they had received. There was no significant difference between the cases and controls in the proportion that had received one or more transplant in the past (*p* = 0.27). There was a variety of diagnoses that served as the primary indication for HSCT, though acute leukemias and neuroblastoma were the most prevalent indications for HSCT. Other indications only accounted for 13% of patients. Interestingly, there was a difference in the distribution of transplant indications seen between cases and controls, with ALL being the most prevalent transplant indication for HSCT in the case group (*p* = 0.02). Radiation was performed as a part of the preparative regimen in the ALL patients with a similar frequency between the cases and controls. Most patients had a myeloablative preparative regimen with no significant difference between cases and controls in the preparative regimen type (*p* = 1.00).

None of the included patients had evidence of pulmonary hypertension based on the baseline pre-HSCT echocardiogram. In addition, all the included patients had a baseline ejection fraction (EF) greater than or equal to 50% by echocardiogram. All baseline echocardiograms were performed within 32 days prior to transplant. The baseline eGFR was largely normal (>60 mL/min/1.73 m^2^) in most included patients (60, 97%) with no significant difference in eGFR found between cases and controls (*p* = 1.00).

### 3.2. Endotheliopathy Data

Of the 31 cases that were diagnosed with endotheliopathy in the first 100 days following HSCT, 22 (71%) had SOS, 8 (26%) had TMA, and 1 (3%) had DAH (Table 2).

The median day of diagnosis of endotheliopathy was 15 days following HSCT (range 6 to 85 days following HSCT). The median near-event ejection fraction by echocardiogram was normal at 70% for the cases and controls (*p* = 0.57). Only two patients had near-event echocardiograms with an EF of 45% in the case group. The rest of the patients had an EF greater than or equal to 50%. There were two patients in the case group and one patient in the control group with near-event echocardiograms showing pulmonary hypertension.

### 3.3. NT-proBNP Levels

**Figure 2:**
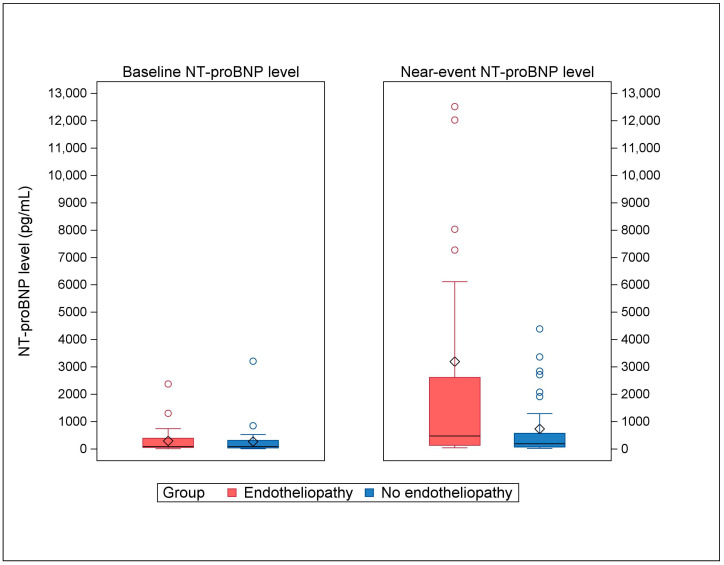
Baseline and near-event NT-proBNP levels in HSCT patients with endotheliopathy compared to the controls. The baseline NT-proBNP levels between cases (endotheliopathy) and controls (no endotheliopathy) were comparable (*p* = 0.51, Wilcoxon rank–sum test). The near-event NT-proBNP levels were elevated in cases compared to controls (*p* = 0.03, Wilcoxon rank–sum test). Horizontal black lines: medians. Diamonds: means. Circles: outliers (one far outlier with the near-event NT-proBNP level of 36,875 pg/mL in the case group is not shown in the figure).

Near-event NT-proBNP levels were significantly elevated in the cases compared to the controls at the most similar timepoint (median 473 pg/mL vs. 187 pg/mL, *p* = 0.03), as displayed in Table 2 and Figure 2. In contrast, the baseline NT-proBNP levels were comparable between the cases and controls, with a median in both groups of 86 pg/mL (*p* = 0.51), as displayed Table 2 and Figure 2.

To confirm differences in the timing of NT-proBNP levels compared to the time of the event in the cases not introducing another variable, we performed an analysis of the timing of the near-event NT-proBNP levels. On average, the difference between the day of diagnosis of endotheliopathy and the day of the near-event NT-proBNP level was 2.84 days, with a standard deviation of 2.79 days (range −2 to 10). Only 2 patients out of 30 cases had a difference of −2 (two days after diagnosis of endotheliopathy). In other words, on average, the near-event NT-proBNP levels in the case group occurred between 2 and 3 days prior to diagnosis of endotheliopathy.

### 3.4. Conditional Logistic Regression

Based on the association of elevated near-event NT-proBNP levels and endotheliopathy, we further evaluated the effect of near-event NT-proBNP levels as well as other variables on the odds of developing endotheliopathy using a conditional logistic regression. None of the variables tested in the primary model, including the near-event NT-proBNP level, baseline NT-proBNP level, near-event EF, baseline EF, and prior transplant(s), had a statistically significant association with the odds of developing endotheliopathy (Table 3).

**Figure 3:**
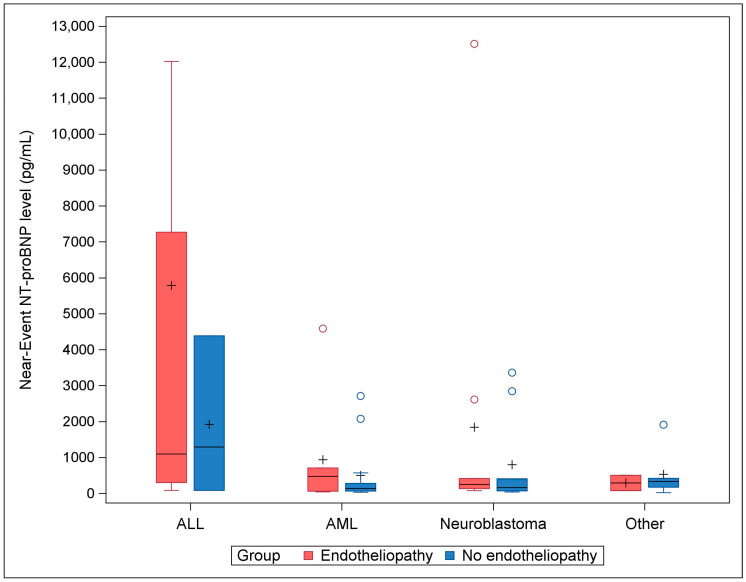
Near-event NT-proBNP levels by transplant indication in patients with and without endotheliopathy. There were 31 cases of endotheliopathy and 31 controls (no endotheliopathy). From left to right: ALL (13 cases and 3 controls), AML (7 cases and 13 controls), neuroblastoma (9 cases and 9 controls), and all other indications (2 cases and 6 controls). Horizontal black lines: medians. Pluses: means. Circles: outliers (one far outlier in the ALL case group with near-event NT-proBNP level of 36,875 pg/mL is not shown in the figure). Abbreviations: ALL, acute lymphoblastic leukemia; AML, acute myeloid leukemia.

Given the unexpected finding of a difference in transplant indication distribution observed between the cases and controls, we explored the effect of transplant indication on the development of endotheliopathy utilizing the secondary model (Table 3). In the secondary model, the estimated effect of transplant indication on developing endotheliopathy was significant, controlling for other covariates (OR = 20.16 with a 95% CI of 1.90 to 213.56, *p* = 0.01). In other words, a transplant indication of ALL increased the odds of developing endotheliopathy 20.16 times compared to the other indications in our study sample. The effect of transplant number was also noteworthy (OR = 6.36 with a 95% CI of 0.95 to 42.48, *p* = 0.06). However, the estimated effect of near-event NT-proBNP was not significant (OR = 1.09 with a 95% CI of 0.83 to 1.43, *p*-value = 0.54) after controlling for the covariates in this model. Nevertheless, as seen in Figure 3, the common trends from the most common transplant indication categories (ALL, AML, and neuroblastoma) suggested an association between an elevated near-event NT-proBNP level and endotheliopathy.

## 4. Discussion

The identification of non-invasive and readily obtainable biomarkers for endotheliopathy in pediatric and young adult HSCT is imperative to facilitate earlier diagnosis and intervention, and eventual improved outcomes. We posit that NT-proBNP should be studied further as a potential biomarker for endotheliopathy in pediatric and young adult patients undergoing HSCT. In our study, while the baseline NT-proBNP levels were found to be comparable between the cases and controls, the near-event NT-proBNP levels were elevated in pediatric and young adult patients diagnosed with endotheliopathy within the first 100 days following HSCT when compared to the matched controls without endotheliopathy. This novel finding has not been previously reported in pediatric and young adult HSCT patients.

Due to small numbers, our study possibly lacked the statistical power to uncover an association between elevated NT-proBNP levels and the development of endotheliopathy following HSCT in a multifactorial logistic regression model. Even with small numbers, having multiple transplants remained an independent risk factor for endotheliopathy in the secondary model.

An interesting and unexpected finding was the difference in distribution of transplant indications seen between the endotheliopathy group and the group without endotheliopathy. Specifically, there was a higher proportion of patients with a primary HSCT indication of ALL in the patients with endotheliopathy compared to those without endotheliopathy. Furthermore, the odds of developing endotheliopathy increased by over 20 times for ALL versus all other diagnoses in the secondary model. A visual inspection of Figure 3 suggests that the effect of NT-proBNP on developing endotheliopathy might be heightened in ALL patients compared to the other diagnoses.

We speculate that ALL patients likely received intensive chemotherapy before HSCT, rendering them potentially more susceptible to both endothelial damage and elevated NT-proBNP levels. Several prospective studies in adult ALL patients undergoing HSCT have demonstrated persistent elevation in NT-proBNP levels in some patients even in absence of clinically significant cardiac symptoms [15,21]. One of these studies also showed persistent NT-proBNP elevation in patients with higher cumulative dose of anthracycline and preparative regimens with total body irradiation or higher dose of cyclophosphamide [21]. A rising NT-proBNP level, particularly in a patient undergoing HSCT for ALL, may suggest that further screening for endotheliopathy is warranted.

Our study had several limitations. Being a single-center study, our numbers were too small to make broad generalizations about NT-proBNP as a biomarker of endotheliopathy in all pediatric and young adult HSCT patients. Being retrospective in nature, we were unable to fully control or standardize the timing of the NT-proBNP level measurements. This was mitigated as much as possible by obtaining retrospective NT-proBNP levels that were temporally very close to the diagnosis of endotheliopathy. Although our study found, on average, the difference between the day of diagnosis of endotheliopathy and the day of near-event NT-proBNP level was only 2.8 days, it would be interesting and informative to see trends in NT-proBNP levels prior to endotheliopathy diagnoses in a prospective study. Additionally, reliance on retrospectively identifying the adverse events can also introduce variability in terms of the timing and sensitivity of DAH, SOS, and TMA diagnoses. While all patients undergoing HSCT at our institution were screened for endotheliopathy, more subtle cases may have been missed in the absence of a prospective trial deliberately screening for them. Lastly, due to our already limited numbers, we were not able to match for disease type (such as ALL). However, in future studies, if the numbers allow for it, matching for disease type would be interesting.

The findings of our study suggest a potential association between elevated NT-proBNP levels and endotheliopathy, particularly in patients undergoing HSCT for acute lymphoblastic leukemia (ALL). To the best of our knowledge, this is the first instance where NT-proBNP has been associated with endotheliopathy syndromes in pediatric and young adult patients undergoing hematopoietic stem cell transplantation (HSCT). NT-proBNP is a laboratory test that is widely available and cost-effective in most clinical settings, aligning with the characteristics of an ideal biomarker [33]. If NT-proBNP could be utilized in conjunction with other clinical symptoms or laboratory tests for the early detection of endotheliopathy, patients stand to benefit from prompt diagnosis and intervention. The use of NT-proBNP as a biomarker for endotheliopathy in pediatric and young adult HSCT patients needs further validation and exploration in future studies, including multi-center and prospective investigations.

## Figures and Tables

**Table 1:** Clinical characteristics of patients with endotheliopathy.

Characteristic	Cases of Endotheliopathy *N* (%)	Controls *N* (%)	Total *N* (%)	*p*-Value (Pearson Exact Chi-Square)
Total patients in cohort	31 (100)	31 (100)	62 (100)	N/A
Age Group				1.00
<2 years	3 (10)	3 (10)	6 (10)	
2–10 years	22 (71)	22 (71)	44 (71)	
>10 years	6 (19)	6 (19)	12 (19)	
Sex				0.44
Female	15 (48)	11 (35)	26 (42)	
Male	16 (52)	20 (65)	36 (58)	
Transplant Type				1.00
Autologous	10 (32)	10 (32)	20 (32)	
Haploidentical	12 (39)	12 (39)	24 (39)	
Matched sibling donor	3 (10)	3 (10)	6 (10)	
Matched unrelated donor	6 (19)	6 (19)	12 (19)	
Transplant Indication				0.02 *
ALL	13 (42)	3 (10)	16 (26)	
AML	7 (23)	13 (42)	20 (32)	
Neuroblastoma	9 (29)	9 (29)	18 (29)	
All Other (Not ALL, AML, Neuroblastoma)	2 (6)	6 (19)	8 (13)	
Congenital neutropenia/MDS derived leukemia	0 (0)	1 (3)	1 (2)	
Familial HLH	0 (0)	1 (3)	1 (2)	
Germ cell tumor	1 (3)	1 (3)	2 (3)	
MDS	0 (0)	1 (3)	1 (2)	
Non-hodgkin lymphoma	0 (0)	1 (3)	1 (2)	
Other acute leukemia	0 (0)	1 (3)	1 (2)	
Sickle cell disease	1 (3)	0 (0)	1 (2)	
Prior Transplant(s)				0.27
0	19 (61)	24 (77)	43 (69)	
≥1	12 (39)	7 (23)	19 (31)	
Preparative Regimen				1.00
Myeloablative	18 (58)	18 (58)	36 (58)	
Non-myeloablative	0 (0)	1 (3)	1 (2)	
Reduced intensity	13 (42)	12 (39)	25 (40)	
Baseline eGFR (mL/min/1.73 m^2^)				1.00
>60	30 (97)	30 (97)	60 (97)	
30–60	1 (3)	0 (0)	1 (2)	
<30	0 (0)	1 (3)	1 (2)	
Baseline presence of pHTN by Echocardiogram				N/A
No	31 (100)	31 (100)	62 (100)	
Baseline EF by Echocardiogram				N/A
≥50%	31 (100)	31 (100)	62 (100)	

* The *p*-value of 0.02 is for comparing the four diagnosis categories (ALL, AML, neuroblastoma, and other) between the cases and the controls. The *p*-value of 0.04 is for the 10 diagnosis categories. Abbreviations: ALL, acute lymphoblastic leukemia; AML, acute myeloid leukemia; MDS, myelodysplastic syndrome; HLH, hemophagocytic lymphohistiocytosis; other acute leukemia was of ambiguous lineage; eGFR, estimated glomerular filtration rate; pHTN, pulmonary hypertension; EF, ejection fraction.

**Table 2:** Endotheliopathy Data.

Characteristic	Cases of Endotheliopathy	Controls	Total	*p*-Value *
Endotheliopathy type, *N* (%)				<0.0001
DAH	1 (3)	0 (0)	1 (2)	
SOS	22 (71)	0 (0)	22 (35)	
TMA	8 (26)	0 (0)	8 (13)	
Not applicable	0 (0)	31 (100)	31 (50)	
Days following HSCT of endotheliopathy diagnosis				N/A
Median (range)	15 (6, 85)	N/A	N/A	
Near-event NT-proBNP level in pg/mL				0.03
Mean (standard deviation)	3190.2 (7151.8)	729.7 (1158.4)	1959.9 (5230)	
Median (range)	473 (40, 36875)	187 (18, 4385)	262.5 (18, 36875)	
Baseline NT-proBNP level in pg/mL				0.51
Mean (standard deviation)	292.1 (477.3)	267.1 (577.8)	279.6 (525.7)	
Median (range)	86 (6, 2373)	86 (5, 3205)	86 (5, 3205)	
Days following HSCT of near-event NT-proBNP level collected				0.25
Median (range)	13 (5, 84)	12 (4, 76)	12.5 (4, 84)	
Near-event EF in percent by echocardiogram				0.57
Median (range)	70 (45, 80)	70 (50, 75)	70 (45, 80)	
Near-event presence of pHTN by echocardiogram, N (%)				1.00
No	29 (94)	30 (97)	59 (95)	
Yes	2 (6)	1 (3)	3 (5)	

* For categorical variables, *p*-values were derived from the exact Pearson Chi-Square test. For numeric variables, the *p*-values were derived from the Wilcoxon rank–sum test. Abbreviations: DAH, diffuse alveolar hemorrhage; SOS, sinusoidal obstruction syndrome; TMA, thrombotic microangiopathy; EF, ejection fraction; pHTN, pulmonary hypertension.

**Table 3:** Estimated odds ratios of development of endotheliopathy.

	Variable	Unit	Odds Ratio * (95% CI)	*p*-Value
Primary Model	Baseline NT-proBNP level	1000 pg/mL	1.08 (0.26–4.51)	0.91
AIC = 58.25	Baseline EF	10%	0.31 (0.07–1.39)	0.12
	Near-event NT-proBNP level	1000 pg/mL	1.32 (0.94–1.85)	0.11
	Near-event EF	10%	2.51 (0.90–7.06)	0.08
	Prior transplant(s)	1 Transplant	2.83 (0.54–14.77)	0.22
Secondary Model	Baseline NT-proBNP level	1000 pg/mL	0.44 (0.07–2.76)	0.38
AIC = 52.57	Baseline EF	10%	0.34 (0.06–1.86)	0.21
	Near-event NT-proBNP level	1000 pg/mL	1.09 (0.83–1.43)	0.54
	Near-event EF	10%	2.32 (0.65–8.32)	0.20
	Prior transplant(s)	1 Transplant	6.36 (0.95–42.48)	0.06
	Age difference	1 year	0.75 (0.52–1.07)	0.11
	Transplant indication of ALL vs. all other indications	N/A	20.16 (1.90–213.56)	0.01

* The odds ratios of development of endotheliopathy and their 95% confidence intervals were estimated using every one-unit increase in each numeric variable, as specified in the unit column. Abbreviations: EF, ejection fraction; ALL, acute lymphoblastic leukemia; AIC, Akaike information criterion.

## Data Availability

The data presented in this study are available only on request from the corresponding author due to the privacy of participants/patients.

## References

[1] Ahmad A.H., Mahadeo K.M. (2021). Perspective: A Framework to Screen Pediatric and Adolescent Hematopoietic Cellular Therapy Patients for Organ Dysfunction: Time for a Multi-Disciplinary and Longitudinal Approach. Front. Oncol..

[2] Carreras E., Diaz-Ricart M. (2011). The Role of the Endothelium in the Short-Term Complications of Hematopoietic SCT. Bone Marrow Transplant..

[3] Pagliuca S., Michonneau D., Sicre de Fontbrune F., Sutra del Galy A., Xhaard A., Robin M., Peffault de Latour R., Socie G. (2019). Allogeneic Reactivity–Mediated Endothelial Cell Complications after HSCT: A Plea for Consensual Definitions. Blood Adv..

[4] Cooke K.R., Jannin A., Ho V. (2008). The Contribution of Endothelial Activation and Injury to End-Organ Toxicity Following Allogeneic Hematopoietic Stem Cell Transplantation. Biol. Blood Marrow Transplant..

[5] Hildebrandt G.C., Chao N. (2020). Endothelial Cell Function and Endothelial-related Disorders Following Haematopoietic Cell Transplantation. Br. J. Haematol..

[6] Lia G., Giaccone L., Leone S., Bruno B. (2021). Biomarkers for Early Complications of Endothelial Origin After Allogeneic Hematopoietic Stem Cell Transplantation: Do They Have a Potential Clinical Role?. Front. Immunol..

[7] Zinter M.S., Dvorak C.C., Spicer A., Cowan M.J., Sapru A. (2015). New Insights into Multicenter PICU Mortality Among Pediatric Hematopoietic Stem Cell Transplant Patients*. Crit. Care Med..

[8] Zaidman I., Mohamad H., Shalom L., Ben Arush M., Even-Or E., Averbuch D., Zilkha A., Braun J., Mandel A., Kleid D. (2022). Survival of Pediatric Patients Requiring Admission in the Intensive Care Unit Post Hematopoietic Stem Cell Transplantation: Prognostic Factors Associated with Mortality. Pediatr. Blood Cancer.

[9] Rubattu S., Sciarretta S., Valenti V., Stanzione R., Volpe M. (2008). Natriuretic Peptides: An Update on Bioactivity, Potential Therapeutic Use, and Implication in Cardiovascular Diseases. Am. J. Hypertens..

[10] Chung T., Lim W.-C., Sy R., Cunningham I., Trotman J., Kritharides L. (2008). Subacute Cardiac Toxicity Following Autologous Haematopoietic Stem Cell Transplantation in Patients with Normal Cardiac Function. Heart.

[11] Masuko M., Ito M., Kurasaki T., Yano T., Takizawa J., Toba K., Aoki S., Fuse I., Kodama M., Furukawa T. (2007). Plasma Brain Natriuretic Peptide during Myeloablative Stem Cell Transplantation. Intern. Med..

[12] Roziakova L., Bojtarova E., Mistrik M., Dubrava J., Gergel J., Lenkova N., Mladosievicova B. (2012). Serial Measurements of Cardiac Biomarkers in Patients after Allogeneic Hematopoietic Stem Cell Transplantation. J. Exp. Clin. Cancer Res..

[13] Horacek J.M., Tichy M., Pudil R., Jebavy L., Zak P., Ulrychova M., Slovacek L., Maly J. (2008). Multimarker Approach to Evaluation of Cardiac Toxicity during Preparative Regimen and Hematopoietic Cell Transplantation. Neoplasma.

[14] Snowden J., Hill G., Hunt P., Carnoutsos S., Spearing R., Espiner E., Hart D. (2000). Assessment of Cardiotoxicity during Haemopoietic Stem Cell Transplantation with Plasma Brain Natriuretic Peptide. Bone Marrow Transplant..

[15] Roziakova L., Bojtarova E., Mistrik M., Krajcovicova I., Mladosievicova B. (2012). Abnormal Cardiomarkers in Leukemia Patients Treated with Allogeneic Hematopoietic Stem Cell Transplantation. Bratisl. Med. J..

[16] Se Z., Zhou H., Li H., Sun J., Zhan Q., Zeng Q., Liu Q., Xu D. (2020). Clinical Characteristics of Patients with Different N-Terminal Probrain Natriuretic Peptide Levels after Hematopoietic Stem Cell Transplantation. Dis. Markers.

[17] Roziakova L., Mistrik M., Batorova A., Kruzliak P., Bojtarova E., Dubrava J., Gergel J., Mladosievicova B. (2015). Can We Predict Clinical Cardiotoxicity with Cardiac Biomarkers in Patients After Haematopoietic Stem Cell Transplantation?. Cardiovasc. Toxicol..

[18] Niwa N., Watanabe E., Hamaguchi M., Kodera Y., Miyazaki H., Kodama I., Ohono M. (2001). Early and Late Elevation of Plasma Atrial and Brain Natriuretic Peptides in Patients after Bone Marrow Transplantation. Ann. Hematol..

[19] Rotz S.J., Ryan T.D., Hlavaty J., George S.A., El-Bietar J., Dandoy C.E. (2017). Cardiotoxicity and Cardiomyopathy in Children and Young Adult Survivors of Hematopoietic Stem Cell Transplant. Pediatr. Blood Cancer.

[20] Rotz S.J., Dandoy C.E., Taylor M.D., Jodele S., Jefferies J.L., Lane A., El-Bietar J.A., Powell A.W., Davies S.M., Ryan T.D. (2017). Long-Term Systolic Function in Children and Young Adults after Hematopoietic Stem Cell Transplant. Bone Marrow Transplant..

[21] Horacek J.M., Pudil R., Tichy M., Jebavy L., Zak P., Slovacek L., Maly J. (2007). Biochemical Markers and Assessment of Cardiotoxicity During Preparative Regimen and Hematopoietic Cell Transplantation in Acute Leukemia. Exp. Oncol..

[22] Zaucha-Prażmo A., Sadurska E., Drabko K., Kowalczyk J.R. (2016). Can We Find a Good Biochemical Marker of Early Cardiotoxicity in Children Treated with Haematopoietic Stem Cell Transplantation?. Współczesna Onkol..

[23] Wu P., Huo W., Zhao H., Lv J., Lv S., An Y. (2024). Risk Factors and Predictive Model for Mortality in Patients Undergoing Allogeneic Hematopoietic Stem Cell Transplantation Admitted to the Intensive Care Unit. Exp. Ther. Med..

[24] McArthur J., Fitzgerald J., Bajwa R., Margossian S., Yates A., Duncan C., Talano J., Spear D., Luther K., Tamburro R. (2012). 763: Brain Natriuretic Peptide (Bnp) Levels as a Predictor of Need for Critcal Care Resources in Pediatric Hematopoietic Stem Cell Transplant (HSCT) Patients. Crit. Care Med..

[25] Kim D.H., Ha E.J., Park S.J., Koh K.-N., Kim H., Im H.J., Jhang W.K. (2021). Prognostic Factors of Pediatric Hematopoietic Stem Cell Transplantation Recipients Admitted to the Pediatric Intensive Care Unit. Acute Crit. Care.

[26] Fu S., Ping P., Wang F., Luo L. (2018). Synthesis, Secretion, Function, Metabolism and Application of Natriuretic Peptides in Heart Failure. J. Biol. Eng..

[27] Ma K.K., Ogawa T., De Bold A.J. (2004). Selective Upregulation of Cardiac Brain Natriuretic Peptide at the Transcriptional and Translational Levels by Pro-Inflammatory Cytokines and by Conditioned Medium Derived from Mixed Lymphocyte Reactions via P38 MAP Kinase. J. Mol. Cell. Cardiol..

[28] Kuhn M. (2012). Endothelial Actions of Atrial and B-type Natriuretic Peptides. Br. J. Pharmacol..

[29] Hall C. (2005). NT-ProBNP: The Mechanism Behind the Marker. J. Card. Fail..

[30] Kataoka K., Nannya Y., Iwata H., Seo S., Kumano K., Takahashi T., Nagai R., Kurokawa M. (2010). Plasma Brain Natriuretic Peptide Is Associated with Hepatic Veno-Occlusive Disease and Early Mortality after Allogeneic Hematopoietic Stem Cell Transplantation. Bone Marrow Transplant..

[31] Corbacioglu S., Carreras E., Ansari M., Balduzzi A., Cesaro S., Dalle J.-H., Dignan F., Gibson B., Guengoer T., Gruhn B. (2018). Diagnosis and Severity Criteria for Sinusoidal Obstruction Syndrome/Veno-Occlusive Disease in Pediatric Patients: A New Classification from the European Society for Blood and Marrow Transplantation. Bone Marrow Transplant..

[32] Jodele S., Davies S.M., Lane A., Khoury J., Dandoy C., Goebel J., Myers K., Grimley M., Bleesing J., El-Bietar J. (2014). Diagnostic and Risk Criteria for HSCT-Associated Thrombotic Microangiopathy: A Study in Children and Young Adults. Blood.

[33] Navanandan N., Searns J., Ambroggio L. (2023). Method/Ology of Phases of Biomarker Discovery. Hosp. Pediatr..

